# Supplementary material for: Multi‐dataset identification of innovative feature genes and molecular mechanisms in keratoconus
Source: J Cell Mol Med. 2024 Sep 19;28(18):e70079. doi: 10.1111/jcmm.70079 (PMC11412914; doi:10.1111/jcmm.70079)
Supplement: Supplementary file 2 — Material S2. [file JCMM-28-e70079-s002.zip › 7.GSEA analysis/5.RNF217-AS1/KEGG_STEROID_BIOSYNTHESIS.html]

Details for gene set KEGG\_STEROID\_BIOSYNTHESIS[GSEA]

|  || Dataset | RNF217-AS1.group.cls#h\_versus\_l |
| Phenotype | group.cls#h\_versus\_l |
| Upregulated in class | h |
| GeneSet | KEGG\_STEROID\_BIOSYNTHESIS |
| Enrichment Score (ES) | 0.2762067 |
| Normalized Enrichment Score (NES) | 0.8571969 |
| Nominal p-value | 0.62376237 |
| FDR q-value | 1.0 |
| FWER p-Value | 1.0 |
Table: GSEA Results Summary

  

Fig 1: Enrichment plot: KEGG\_STEROID\_BIOSYNTHESIS      
 Profile of the Running ES Score & Positions of GeneSet Members on the Rank Ordered List

  

| SYMBOL | TITLE | RANK IN GENE LIST | RANK METRIC SCORE | RUNNING ES | CORE ENRICHMENT || 1 | CYP27B1 | na | 3568 | 0.269 | 0.0787 | Yes |
| 2 | CEL | na | 4655 | 0.229 | 0.2020 | Yes |
| 3 | MSMO1 | na | 7340 | 0.143 | 0.2216 | Yes |
| 4 | HSD17B7 | na | 9457 | 0.096 | 0.2255 | Yes |
| 5 | SOAT2 | na | 10031 | 0.086 | 0.2671 | Yes |
| 6 | SQLE | na | 11271 | 0.066 | 0.2762 | Yes |
| 7 | CYP51A1 | na | 14205 | 0.028 | 0.2108 | No |
| 8 | DHCR7 | na | 15043 | 0.019 | 0.1992 | No |
| 9 | LSS | na | 15550 | 0.014 | 0.1938 | No |
| 10 | SC5D | na | 15994 | 0.009 | 0.1869 | No |
| 11 | NSDHL | na | 16084 | 0.008 | 0.1897 | No |
| 12 | EBP | na | 17044 | -0.002 | 0.1635 | No |
| 13 | FDFT1 | na | 19890 | -0.029 | 0.1010 | No |
| 14 | DHCR24 | na | 21540 | -0.046 | 0.0843 | No |
| 15 | LIPA | na | 23664 | -0.070 | 0.0707 | No |
| 16 | TM7SF2 | na | 25608 | -0.100 | 0.0824 | No |
| 17 | SOAT1 | na | 31571 | -0.267 | 0.0909 | No |
Table: GSEA details [plain text format]

  

Fig 2: KEGG\_STEROID\_BIOSYNTHESIS      
 Blue-Pink O' Gram in the Space of the Analyzed GeneSet

  

Fig 3: KEGG\_STEROID\_BIOSYNTHESIS: Random ES distribution      
 Gene set null distribution of ES for **KEGG\_STEROID\_BIOSYNTHESIS**
